# Enzyme-assisted modification of flavonoids from *Matricaria chamomilla*: antioxidant activity and inhibitory effect on digestive enzymes

**DOI:** 10.1080/14756366.2019.1681989

**Published:** 2019-10-28

**Authors:** Elida Paula Dini de Franco, Fabiano Jares Contesini, Bianca Lima da Silva, Anna Maria Alves de Piloto Fernandes, Camila Wielewski Leme, João Pedro Gonçalves Cirino, Paula Renata Bueno Campos, Patrícia de Oliveira Carvalho

**Affiliations:** a Laboratory of Multidisciplinary Research, São Francisco University (USF), Bragança Paulista, Brazil; b Department of Biochemistry and Tissue Biology, Institute of Biology, State University of Campinas (UNICAMP), Campinas, Brazil; c Department of Biochemistry, Institute of Biology, State University of Campinas (UNICAMP), Campinas, Brazil

**Keywords:** *Matricaria chamomilla*, flavonoids, hesperidinase, β-galactosidase

## Abstract

*Matricaria chamomilla* L. contains antioxidant flavonoids that can have their bioactivity enhanced by enzymatic hydrolysis of specific glycosyl groups. This study implements an untargeted metabolomics approach based on ultra-performance liquid chromatography coupled with electrospray ionisation quadrupole time-of-flight mass spectrometry technique operating in MS^E^ mode (UPLC-QTOF-MS^E^) and spectrophotometric analysis of chamomile aqueous infusions, before and after hydrolysis by hesperidinase and β-galactosidase. Several phenolic compounds were altered in the enzymatically treated infusion, with the majority being flavonoid derivatives of apigenin, esculetin, and quercetin. Although enzymatically modifying the infusion only led to a small increase in antioxidant activity (DPPH**•** method), its inhibitory effect on pancreatic lipase was of particular interest. The enzymatically treated infusion exhibited a greater inhibitory effect (EC_50_ of 35.6 µM) than unmodified infusion and kinetic analysis suggested mixed inhibition of pancreatic lipase. These results are of great relevance due to the potential of enzymatically treated functional foods in human health.

## Introduction

*Matricaria chamomilla* L. (i.e. chamomile), native to southern and eastern Europe and widely found in Brazil, is one of the oldest known herbs of traditional medicine and belongs to the Asteraceae family. It contains a large group of therapeutically interesting and active compounds, the main classes being the flavonoids, sesquiterpenes, coumarins, and polyacetylenes^1^. The flowers in particular, contain a large amount of hydrophilic constituents (sugars, flavonoids, mucilages, phenyl carbonic acids, amino acids, choline, salts)^2^. Flavonoids such as apigenin-7-O-glucoside, apigenin, luteolin-7-O-glucoside and luteolin (flavones), rutin and quercetin (flavonols) and many other phenolic compounds such as herniarin and umbelliferone (coumarin), chlorogenic acid, and caffeic acid (phenylpropanoids) are found in chamomile extract^3^. Among those, apigenin and esculin are the most promising compounds with respect to human health. They are present in very small quantities as free apigenin and esculetin, respectively, but predominantly exist in the form of various glycosides^4^.

Polyphenols are secondary metabolites affected by environmental and seasonal conditions and by the geographic origin of plants. In that light, a diverse polyphenolic fingerprint characterises the *Matricaria chamomilla* L. plant and, evidently, its biological activity too. Chamomile is used mainly as an anti-inflammatory and antiseptic, but also has anticonvulsant, antispasmodic, and analgesic properties. *In vitro* and *in vivo* studies have found that *M. chamomilla* L. exhibits antifungal, antihypertensive, antiallergic, hypoglycaemic, analgesic, immunomodulatory, antiulcerogenic, hepatoprotective, chemopreventive, and anticancer properties^2^. Chamomile may also have sedative and anxiolytic effects because of apigenin’s ability to bind to benzodiazepine receptors potentiating the activity at GABA A receptors^5^.

Natural flavonoids, predominantly found in their glycosylated form, are widely known for their antioxidant potential^6^^,^^7^ and as digestive enzyme inhibitors^8–11^. Their antioxidant activity depends on the position and structure of the sugar(s) in the flavonoid molecule and usually decreases with the increase in the number of glycosidic moieties linked to a hydrophobic aglycone. Glycosylated flavonoids generally display lower antioxidant capacity compared to the corresponding aglycones^7^^,^^12^^,^^13^.

The hydrolysis of the glycosidic fractions of various natural flavonoids employing specific enzymes is an excellent way to modify the structure and improve the physicochemical and biological properties of flavonoids^14^. Previous reports have shown that the hydrolysis of specific glycosyl groups of glycosylated flavonoids increases the antioxidant and antiproliferative activities of rutin^12^ and increases the antioxidant activity of kaempferol^15^, besides improving the anti-inflammatory activity of naringin^16^. The use of enzymes has been of great scientific and industrial interest due to their wide availability, high selectivity and their promotion of efficient reactions with few by-products.

Commercial hesperidinase and β-galactosidase are widely employed in biotechnological deglycosylation processes producing the respective partially deglycosylated flavonoids or aglycones. Hesperidinase is a fungal enzyme that expresses both α-l-rhamnosidase (EC 3.2.1.40) and β-d-glucosidase (3.2.1.21) activities resulting in rhamnose and glucose release, respectively, from a variety of conjugated flavonoids^17^. β-galactosidase can be obtained in large quantities from a special strain of *Aspergillus oryzae* and exhibits a strong linkage specificity for β 1–4 linkages galactosyl residues^18^. Previous results have shown the feasibility of producing highly purified kaempferol from two kaempferol glycosides by enzymatic hydrolysis using the optimum combination of the enzymes hesperidinase and β-galactosidase^15^. The combination of these two enzymes may account for a significant metabolic change in plant extracts rich in conjugated flavonoids such as chamomile infusion.

In the present work, the enzymatic hydrolysis of an aqueous infusion of *Matricaria chamomilla* L. was carried out using the combination of these enzymes (hesperidinase and β-galactosidase) and subsequently the metabolite profile, the antioxidant activity and the inhibitory effect on digestive enzymes of native and enzymatically modified infusions were evaluated. To elucidate the discriminating metabolic profile after the enzymatic hydrolysis of the chamomile infusion, an untargeted metabolomics^19^ approach based on UPLC-QTOF-MS^E^ was performed. This high resolution platform integrates full MS with MS/MS fragmentation for all precursor ions simultaneously^20^, allowing high-throughput acquisition of data and simultaneous annotation of diverse groups of secondary metabolites.

## Methods

### Enzymes and reagents

Hesperidinase (Hesperidin-α-1,6-rhamnosidase) from *Penicillium* sp., β-galactosidase from *Aspergillus oryzae*, lipase from porcine pancreas (type II), α-amylase from *Bacillus subtilis*, α-glucosidase from *Bacillus stearothermophilus*, DPPH**•** (2,2-diphenyl-1-picril-hydrozyl radical), nitrophenyl-α-D-glucopyranoside, and p-nitrophenyl palmitate were purchased from Sigma-Aldrich (St. Louis, MO).

### Preparation of the Matricaria chamomilla L.Infusion

The infusion was prepared by crushing dried plant material purchased from a local market in the city of Bragança Paulista, Brazil. A sample of plant material was accurately weighed, added to boiling distilled water (at a 1:10 w/v sample to solvent ratio) in a stainless steel pot and left at 25 °C for 15 min, then filtered under reduced pressure. Prior to analysis, the extraction solution was filtered thought a 0.20 µm nylon membrane filter into a HPLC vial.

### Bioconversion reaction

The bioconversion reaction was carried out in screw-capped glass tubes with shaking (130 rpm) at controlled temperature (40 °C) for 8 h using 10 ml of aqueous infusion of *Matricaria chamomilla*. To initiate the hydrolysis of the flavonoid glycosides, 1 ml of enzyme mixture solution prepared in 0.1 M acetate buffer pH 4.0 was added to the reaction mixture. The enzyme mixture used had equal parts of each up (hesperidinase and β-galactosidase) to a final concentration of 0.02 mg/mL. Optimisation of hydrolysis conditions and incubation time using the enzyme combination was previously performed^21^. The reactions were stopped by boiling for 20 min and the samples were stored in a refrigerator to await analysis. The assays were performed in triplicate. According to the manufacturer’s information, hesperidinase expresses both α-l-rhamnosidase (EC 3.2.1.40) and β-d-glucosidase (3.2.1.21) activities. One unit will liberate 1.0 µmole of reducing sugar (as glucose) from hesperidin per min at pH 3.8, at 40 °C. For β-galactosidase activity, one unit will hydrolyse 1.0 µmole of lactose per minute at pH 4.5, at 30 °C.

### *Composition of Matricaria chamomilla* L. *Infusion by UHPLC-Q-TOF-MS^E^*

The analytical LC/MS experiments were performed on an ACQUITY FTN liquid chromatograph coupled to a XEVO-G2XSQTOF mass spectrometer (Waters, Manchester, NH). Data were acquired using MassLynx software. For liquid chromatography, an Acquity UPLC BEH C18 (2.1 × 50 mm 1.7 µm, Waters) column was used. Water was used as mobile phase A, while acetonitrile was used as mobile phase B in a gradient elution mode. The flow rate was 0.4 ml/min at 45 °C and the gradient composition was 5% of B (initial), 95% B (8.0 min), 95% B (8.5 min), 5% B (8.60 min, with 1.4 min left for column re-equilibration), resulting in a 10 min analysis overall; the injection volume was 0.1 µL. For mass spectrometry, data were acquired in positive and negative modes. For positive mode, electrospray ionisation source parameters were set as follows: capillary voltage of 3000 V, sampling cone of 40 V, source temperature of 150 °C, desolvatation temperature of 450 °C, cone gas flow of 50 L/h, and desolvatation gas flow of 900 L/h. For negative mode: capillary voltage of 2500 V, sampling cone of 40 V, source temperature of 150 °C, desolvation temperature of 450 °C, cone gas flow of 50 L/h, and desolvation gas flow of 900 L/h. The acquisition scan range was from 100 to 1000 Da, and the data were acquired using the MS^E^ approach. Leucine encephalin (molecular mass of 555.62; 200 pg/µL in 1:1 ACN:H_2_O) was used as a lock mass for accurate mass measurements and a 0.5 mmol/L sodium formate solution was used for instrument calibration.

### MS data processing and statistics

Progenesis QI (Waters) was used for peak detection, alignment, deconvolution, data filtering, ion annotation and MS^E^-based putative identification results of LC–MS raw data prior to statistical analysis. PolyPhenols_PubChemID_v2013.24.11.01 database was used for identification with the following search parameters: precursor mass error ≤5 ppm, fragment tolerance ≤10 ppm. Fragmentation score, mass accuracy, and isotope similarity were considered for the identification of the molecules. To find any significant differences between the infusion before and after the enzymatic modification had been performed, univariate statistical analyses were applied using the MetaboAnalyst 4.0 web platform (http://www.metaboanalyst.ca). Data were normalised by sum and pareto scaled before performing statistics. Fold change (FC), T-test, and Volcano plot methods were applied. Only features that fulfilled log2(FC) > 2, *p* values < .05 and FDR < 0.05 were considered significant. Solutions were analysed in quadruplicate.

### DPPH• radical-scavenging activity

The antioxidant activity of samples was assessed on the basis of the scavenging activity of the stable 2, 2-diphenylpicrylhydrazyl free radical (DPPH•). Approximately, 250 µL of a methanolic solution of DPPH**•** (0.02 mg/L) was added to 40 µL of aqueous infusion in acetate buffer 0.3 M, pH 3.8 or methanol, in the case of control. Flasks were incubated at 25 °C for 25 min and absorbance was determined at 517 nm. All assays were performed in triplicate. The scavenging capacity of the DPPH radical was calculated using the following equation:

DPPH• scavenging effect (%) = (Ac – As/Ac) × 100,

where Ac and As are absorbance values of control reaction and test samples, respectively.

### In vitro digestive enzyme inhibition assays

The inhibitory effect of chamomile toward digestive enzymes was evaluated using both native infusion and the enzymatically modified infusion.

The α-amylase activity was determined using starch as substrate, according to the methodology proposed by Kandra et al.^22^. 50 µL of the chamomile infusions and 50 µL of α-amylase (50 units/mg) were pre-incubated for 20 min in a water bath at 37 °C. The starch (1%, m/v) was prepared in phosphate buffer at pH 7.0 with 38 mmol/L NaCl. After the addition of 100 µL of the substrate, the mixture was incubated for 2 h. The product (glucose) was quantified by the glucose oxidase-peroxidase method with a commercial kit (Labtest) and measured at 520 nm. Phosphate buffer at pH 7.0 was used as control.

The inhibitory effect on *α*-glucosidase was tested according to a previously reported protocol^23^, using p-nitrophenyl α-d-glucopyranoside as a substrate. Activity was assayed by using 50 µl of the chamomile infusion and 100 µl α-glucosidase solution (1.0 U/ml in 0.1 M phosphate buffer pH 7.0) incubated in a water bath at 25 °C for 10 min. After preincubation, 50 µl of substrate (5 mM p-nitrophenyl-α-d-glucopyranoside solution in 0.1 M phosphate buffer pH 7.0) were added to each tube at timed intervals. The reaction mixtures were incubated at 25 °C for 5 min. The absorbance readings were recorded at 405 nm and phosphate buffer at pH 7.0 was used as control.

The infusion was analysed for its ability to inhibit lipase activity using a modified method with p-nitrophenyl palmitate as substrate and porcine pancreatic lipase (PL)^24^. The mixture of 100 µL of pancreatic lipase (diluted in 50 mM Tris–HCl buffer pH 8.0, containing 10 mM CaCl_2_ and 25 mM NaCl), 50 µL of the infusion, and 50 µL of 4 mmol/L p-nitrophenyl palmitate (0.05 mmol/L Tris − HCl buffer at pH 8.0) was incubated for 30 min. The reaction was stopped, transferring the tubes to an ice bath and adding 500 µL of 0.05 mmol/L Tris − HCl buffer at pH 8.0. The absorbance readings were recorded at 405 nm and Tris–HCl buffer (pH 8.0) was used as control. The concentration of inhibitors required for inhibiting 50% of the lipase activity under the assay conditions was defined as the EC_50_ value.

All analyses were performed in triplicate and results were presented as percentage (%) of inhibition (I). The results were calculated using the following equation:

$$I\;\left(\%\right)\;=\;\left[\left(A_{\text{control}}–\;A_{\text{sample}}\right)/\left(A_{\text{control}}\right)\right]\;\times \;100,$$

where *A*_control_ is the absorbance value of the negative control and *A*_sample_ is the absorbance value of the chamomile sample.

### Measurement of the kinetic constants

In order to measure the Michaelis–Menten constant, *K*_m_, the inhibition constant, *K*_i_, and the *V*_max_ (maximum reaction rate), a series of substrate concentrations (200–800 mM) was tested in the assay system. Each analysis was performed without (control) and with the chamomile infusion as inhibitor (40 and 60 µl). *V*_max_ and *K*_m_ were calculated and the type of inhibition kinetics was identified using the SigmaPlot software (Aspire Software International, Ashburn, VA). The Michaelis–Menten equation linearised by Lineweaver–Burk was used to determine *V*_max_ and *K*_m_ by plotting a graph, i.e. 1/*V* against 1/[substrate concentration], and estimated by the intercept and slope, respectively.

### Statistical analysis of antioxidant and enzymatic activities

Statistical analyzes and graphs were done using GraphPad Prism 5.0 software. The data were expressed as mean ± standard deviation (SD) and the significance of difference was calculated using one-way ANOVA and Tukey as post-test of multiple comparison. Values of *p* < .05 were considered significant.

## Results

### Metabolic profile

UHPLC-Q-TOF-MS^E^ analysis of the chamomile infusion enabled the putative identification^19^ of more than 100 phenolic compounds that could be classified into flavonoid glycosides and derivatives of hydroxycinnamic acid and coumarins among others. From among the well-known chamomile compounds described in the literature for the native infusion, the following were identified: apigenin derivatives (apigenin-7-apioglucoside, apigenin-7-O-glucoside and apigenin-7-glucuronide), luteoloside, kaempferol glycosides, luteolin, umbelliferone, caffeic acid, rutin, and isoquercetin.

**Figure 1.**
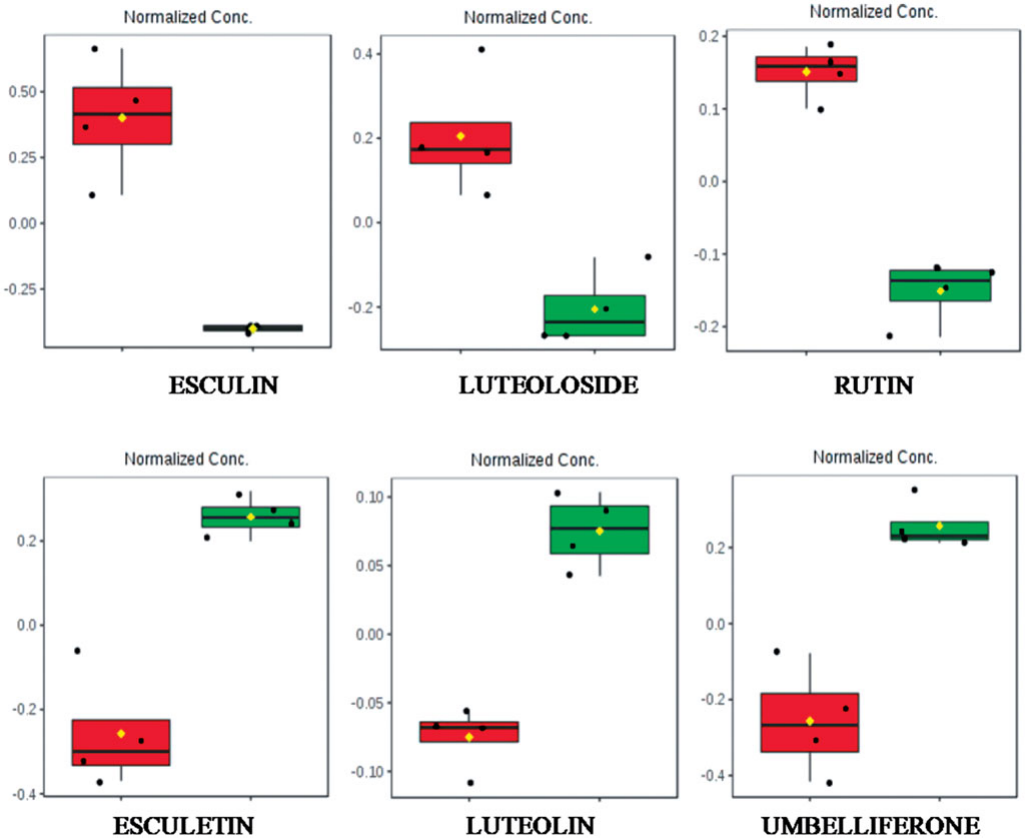
Boxplots of some selected statistically significant (*p* > .05) metabolites from chamomile infusion indicating normalised intensity differences before (red) and after (green) enzymatic treatment. Esculin (1.33_340.0800n), luteoloside (2.44_448.1009n), rutin (2.14_611.1610*m/z*), esculetin (1.71_179.0343*m/z*), luteolin (2.98_287.0552*m/z*) and umbelliferone (0.60_163.0394*m/z*). All features were observed in the positive ionisation mode.

The putative identities of statistically significant features of compounds from the enzymatic treatment of the infusion were investigated by comparing fragmentation pattern data acquired using an MS^E^ approach with the available metabolite database. A wide variety of secondary metabolites were altered with the enzymatic treatment and the main ones are shown in the Table 1 and Figure 1.

**Table 1.** Putative identification of statistically significant secondary metabolites from chamomile infusion and the relative tendency after the enzymatic treatment.

Feature^a^ number	Feature label	*p* Value^b^	FDR	Formula	Mass error^c^ (ppm)	ID^d^	Mode	Trend^e^
1	1.97_165.0548*m/z*	5.5999E-10	1.0685E-08	C9H8O3	1.02	2-Hydroxy Cinnamic Acid (o-coumaric acid)	ESI(+)	High
2	2.46_189.0549*m/z*	2.0257E-09	2.5899E-08	C11H12O5	1.03	4-Hydroxy-3,5-dimethoxycinnamic acid	ESI(+)	High
4	0.50_198.0524n	1.34E-03	1.02E-02	C9H10O5	−2.26	Alpha-(3,4-Dihydroxyphenyl) lactic acid	ESI(−)	High
5	2.67_180.0426n	1.683E-10	5.1599E-09	C9H8O4	1.65	3,4-dihydroxycinnamic acid (caffeic acid)	ESI(+)	High
6	5.13_271.0965*m/z*	1.09E-05	2.07E-05	C16H14O4	0.09	Dihydroformononetin	ESI(+)	High
7	1.26_155.0340*m/z*	1.30E-07	4.87E-07	C7H6O4	1.04	Dihydroxybenzoic acid	ESI(+)	High
8	4.00_182.1178*m/z*	1.3385E-12	5.5759E-10	C10H12O2	1.61	Eugenic acid	ESI(+)	High
9	3.51_301.0709*m/z*	2.8657E-07	9.3267E-07	C16H12O6	0.85	Hispidulin	ESI(+)	High
10	3.21_179.0706*m/z*	3.8202E-10	8.4129E-09	C10H12O4	1.81	Hydroferulic acid	ESI(+)	High
11	2.67_135.0442*m/z*	1.33E-11	1.35E-09	C8H8O3	1.24	Hydroxyphenylacetic acid	ESI(+)	High
12	0.56_169.0134*m/z*	5.37E-04	6.04E-03	C7H6O5	−5.04	Pyrogallolcarboxylic acid (triihydroxybenzoic acid)	ESI(−)	High
13	4.70_343.1175*m/z*	2.13E-06	5.07E-06	C19H18O6	−0.40	Tetramethyl-O-scutellarin	ESI(−)	High
14	3.52_447.1279*m/z*	4.53E-07	1.36E-06	C10H12O5	4.12	Vanillactic acid	ESI(+)	High
15	3.83_184.0370n	1.60E-10	5.08E-09	C8H8O5	−0.80	3-O-Methylgallic acid	ESI(+)	High
16	3.37_474.1166n	1.3749E-08	9.0792E-08	C23H22O11	0.73	6″-O-acetylgenistin	ESI(+)	Low
17	5.48_113.0383*m/z*	9.48E-07	2.55E-06	C9H8O2	−1.87	Cinnamic acid	ESI(+)	Low
18	3.61_303.0137*m/z*	1.14E-06	7.16E-05	C14H10O9	−3.05	Digallic acid	ESI(−)	Low
19	5.48_171.0806*m/z*	1.26E-07	4.76E-07	C12H14O3	0.70	Eugenol acetate	ESI(+)	Low
20	5.48_159.0441*m/z*	1.17E-07	4.50E-07	C10H10O4	0.18	Hesperetic acid (trans-cinnamic acid)	ESI(+)	Low
21	3.35_631.1660*m/z*	6.23E-07	1.77E-06	C28H32O15	4.30	Diosmin (diosmetin 7-rutinoside)	ESI(+)	Low
22	3.83_575.1397*m/z*	4.54E-07	1.36E-06	C27H30O16	0.20	Quercetin 3-O-neohesperidoside	ESI(+)	Low
23	2.84_725.1939*m/z*	1.16E-07	4.47E-07	C32H38O20	2.09	Quercetin 3-(2G-xylosylrutinoside)	ESI(+)	Low
24	1.78_338.1001n	1.07E-10	4.172E-09	C16H18O8	−0.18	4-p-Coumaroylquinic acid	ESI(+)	Low
25	2.33_317.0655*m/z*	1.35E-09	1.9489E-08	C16H12O7	−0.24	6-Methoxyluteolin	ESI(+)	Low
26	3.24_559.1449*m/z*	4.85E-06	1.02E-05	C27H30O15	0.53	Kaempferol-7-neohesperidoside	ESI(+)	Low
27	2.72_591.1348*m/z*	2.25E-09	2.75E-08	C27H30O17	0.63	6,8-Dihydroxykaempferol 3-rutinoside	ESI(+)	Low
28	2.40_535.1087*m/z*	1.01E-08	7.32E-08	C24H22O14	0.87	Luteolin 7-O-(6''-malonylglucoside)	ESI(+)	Low
29	3.65_317.0654*m/z*	9.87E-06	1.90E-05	C16H12O7	−0.61	Isorhamnetin	ESI(+)	Low
30	2.36_519.1142*m/z*	5.84E-08	2.62E-07	C24H22O13	1.63	Malonylgenistin	ESI(+)	Low

^a^Feature: mass-to charge ratio and retention time pairs, ^b^False Discovered Ratio (FDR) adjusted, ^c^Calculated in comparison with theoretical value, ^d^Putative Identity (ID) All listed compounds reached level 2 identification, except for feature numbers 4, 16, 17 and 22 that reached level 3. ^e^In product of hydrolysis when compared to native chamomilla infusion.

Table 1 shows 30 compounds putatively identified by UHPLC-Q-TOF-MS^E^ that were altered with the enzymatic treatment and possibly involved in the antioxidant activity of chamomile infusion. Hydroxylated phenolic acids (such as 2-hydroxy cinnamic acid, 4-hydroxy-3,5-dimethoxycinnamic acid, hydroxybenzoic acid, hydroxyferulic acid, and caffeic acid) and aglycones (hispidulin and hespertin) were increased; while glycosyl isoflavone forms (malonylglycitin and 6″-O-acetylgenistin) and other free phenolic acids (cinnamic and trans-cinnamic acids) were significantly reduced after enzymatic treatment. In addition, LC–MS^E^ putatively identified rutinoside, galactoside, and neohesperidoside of quercetin and kampferol, which had a tendency to reduce due to enzymatic hydrolysis.

Figure 1 shows the boxplot obtained with normalised intensities of altered flavonoids. The glycosylated flavonoids, esculin (esculetin 6-β-d-glucoside), luteoloside (luteolin 7-O-glucoside) and rutin (quercetin, 3-rhamnosyl-glucoside), were reduced, while their aglycones esculetin and luteolin were increased. The aglycone form of rutin (quercetin) has not been identified as a differential metabolite.

In addition, umbelliferone, a natural product of the coumarin family was significantly increased after enzymatic treatment.

### Enzymatic inhibition

Table 2 shows antioxidant activity and the effect of chamomile infusion on digestive enzyme activities in the absence (control) and presence of the inhibitors (infusion before and after enzymatic treatment).

**Table 2.** DPPH**•** scavenging activity (%) and kinetic parameters of inhibitory effect of chamomile infusion toward digestive enzymes activities in the absence (control) and presence of the inhibitors (40 and 60 µM).

	Control	Native chamomile infusion		Enzymatically modified chamomile infusion	
DPPH**•** scavenging activity (%)	–	41.3 ± 3.1^a^		49.5 ± 1.5^b^	
Kinetic parameters		40 µM^1^	60 µM^2^	40 µM^3^	60 µM^4^
α-glucosidase					
Inhibition (%)	0	36.7 ± 8.5^a^	37.4 ± 5.6^a^	45.2 ± 3.1^a^	56.9 ± 3.7^b^
*V*_max_ (µmol/min)	0.79 ± 0.15^a^	0.60 ± 0.29^a^	0.29 ± 0.23 ^a,b^	0.32 ± 0.25^a,b^	0.21 ± 0.08^b^
*K*_m_ (mM)	354.4^a^	332.4^a^	265.2^a^	332.4^a^	295.2^a^
Pancreatic lipase					
Inhibition (%)	0	32.1 ± 6.3^a^	39.2 ± 5.7^a^	60.4 ± 4.9^b^	73.6 ± 3.2^c^
*V*_max_ (µmol/min)	3.19 ± 0.42^a^	3.29 ± 0.63^a^	2.08 ± 0.25^b^	1.97 ± 0.17^c^	1.03 ± 0.04^d^
*K*_m_ (mM)	341.6^a^	756.7^b^	724.5^b^	924.5^c^	1107.3^d^
α-amylase					
Inhibition (%)	0	12.7 ± 4.1^a^	10.8 ± 3.5^a^	9.1 ± 4.8^a^	11.6 ± 6.0^a^
*V*_max_ (µmol/min)	0.43 ± 0.27^a^	0.38 ± 0.14^a^	0.63 ± 0.28^a^	0.45 ± 0.26^a^	0.54 ± 0.14^a^
*K*_m_ (mM)	357.4^a^	312.4^a^	327.9^a^	298.4^a^	350.9^a^

Results are expressed as the mean ± standard deviation (SD) of three determinations. Means within a line with different superscript letters are significantly different *p* < .05.

^1^*R*^2^=0.8294; ^2^*R*^2^=0.9555; ^3^*R*^2^=0.8787; ^4^*R*^2^=0.9025.

The DPPH^●^ method was used to evaluate the hydrogen donating ability, and the antioxidant activity of the samples was expressed as the percentage of DPPH radical-scavenging activity (%). The enzymatic treatment led to an increase of approximately 12% in the antioxidant activity of enzymatically modified infusion in relation to the values observed for native chamomile infusion. A period longer than 8 h of enzymatic treatment showed no apparent change of antioxidant activity (date not shown).

In the α-glucosidase inhibition assay, the modified infusion at 60 µM showed higher inhibitory activity than the native one (56.9 ± 3.7% and 37.4 ± 5.6%, respectively). At the same concentration, lipase inhibition reached 73.6 ± 3.2%, while native infusion only showed 39.2 ± 5.7% of inhibition (*p* = .0008). The response of the modified flavonoids in inhibiting lipase activity is directly proportional to their concentrations and the EC_50_ value was 35.6 µM. With regard to the α-amylase assay, all infusions presented low inhibitory effect both before and after the enzymatic treatment.

At the maximum concentration assessed, the enzymatically modified chamomile infusion exerted a non-significant effect on the kinetics of the reaction catalysed by α-amylase and a small effect on α-glucosidase activity. There was an observable decrease in the *V*_max_ values of 0.79 ± 0.15 to 0.21 ± 0.08 µmol/min of modified infusion and no effect on *K*_m_ in the reaction catalysed by α-glucosidase, using p-nitrophenyl-α-d-glucopyranoside as the substrate, compared to control.

In the assay of pancreatic lipase inhibitory activity with p-nitrophenyl palmitate as the substrate, the presence of modified chamomile infusion at concentrations of 40 µM and 60 µM decreased the *V*_max_ values of 1.97 ± 0.17 to 1.03 ± 0.04 µmol/min, and increased the *K*_m_ value to 924.5 and 1107.3 mM, respectively.

**Figure 2.**
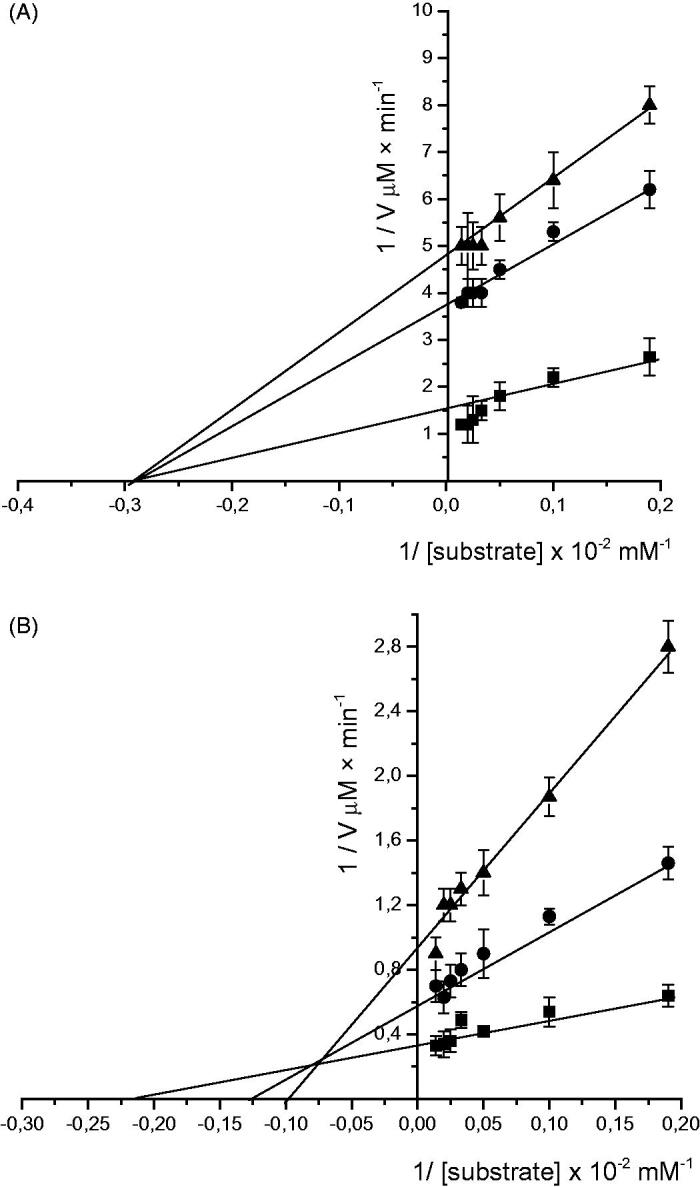
Lineweaver–Burk plot of glucosidase (A) and pancreatic lipase (B) activities. *V* is initial velocity and [*S*] is the concentration of substrate. The values were shown in absence (■) and presence of enzymatically modified chamomile infusion at 40 µM (●) and 60 µM (▲).The values are means of triplicate determinations, and the error bars indicate SD (*n* = 3).

The results were analysed by means of the Lineweaver–Burk double reciprocal plots and the *K*_m_ and *V*_max_ values obtained by graphic extrapolation. The double reciprocal graph was expressed as 1/*V*_o_ (y) plotted against 1/[*S*], where the intercept on the 1/*V*_o_ axis is equal to 1/*V*_max_ and the intercept on the 1/[*S*] axis is equal to −1/*K*_m_ **(**Figure 2).

The alterations of the kinetic parameters suggested two different types of inhibition. On the one hand, in the case of α-glucosidase, the enzymatic inhibition apparently is non-competitive, as we can see a decrease in *V*_max_ and no change in *K*_m_ values. On the other hand, in the pancreatic lipase test, a mixed type of inhibition seems to occur since the value of *V*_max_ increased and *K*_m_ value decreased for both native and enzymatically treated chamomile infusion.

## Discussion

As hypothesised, enzymatic processing altered the chemical composition and biological activity of the chamomile infusion. It is important to emphasise that the untargeted metabolomics approach used here has the principal purpose of elucidating the main statistically validated metabolites discriminating between chamomile infusion and its hydrolysis product rather than providing some kind of chemical characterisation of the native chamomile infusion.

The multicomponent chemical profile of chamomile flowers has been demonstrated previously^1–4^. The main related constituents are cosmosiin (apigenin-7-O-glucopyranoside) and its derivatives [apigenin-7-O-(4″- malonyl)-glucopyranoside, apigenin-7-O-(6″-malonyl)-glucopyranoside, apigenin-7-O-(4″-acetyl)-glucopyranoside, apigenin-7-O-(4″-malonyl-6″-acetyl)-glucopyranoside], phenylpropanoids (3-O- and 3,5-di-O-caffeoylquinic acids, 2-O-glucopyranosides of cis- and trans-2-hydroxy-4-methoxycoumaric acid), coumarins (skimmin, daphnetin, daphnin, umbelliferone, herniarin), as well as terpenes (cis- and trans-spiro-ether).

Both flavonoid and non-flavonoid polyphenols were significantly altered by the acidic enzymatic treatment which resulted in the favourable action of metabolites as inhibitors of digestive enzymes, in particular as inhibitors of pancreatic lipase activity. A significant effect was also observed on glucosidase inhibition. It has been reported that some polyphenols may inhibit digestive enzymes involved in the breakdown of starches and lipids, leading to a reduction in glucose and lipid absorption and therefore to positive effects on obesity and blood glucose control, which is an effective strategy for type 2 diabetes prevention and treatment^25–28^.

Based on the results obtained from UPLC-QTOF-MS^E^ analysis, it could be seen that the enzymatic hydrolysis reaction of polyphenol compounds in the infusion by a combination of glycosyl hydrolases (hesperidinase and galactosidase) led to the increase of aglycone flavonoid levels and of hydroxylated phenolic acids. Derivatives of cinnamic acids (2-hydroxy cinnamic, 3,4-dihydroxycinnamic acid and 4-hydroxy-3,5-dimethoxycinnamic acids) and of benzoic acids (di and trihydroxybenzoic acids) tended to increase after enzymatic treatment compared to their contents in native infusion. Cinnamic acids, especially those with the phenolic hydroxyl group, are well-known antioxidants and are supposed to have several health benefits due to their strong free radical scavenging properties^29^. Chlorogenic acid, the major representative of hydroxycinnamic acids, is an ester formed between quinic acid and caffeic acid (3,4-dihydroxycinnamic acid) and it has been reported that caffeic acid has stronger *in vitro* antioxidant activity than that of chlorogenic acid^30^, which might contribute to the increased antioxidant activity of the modified infusion.

The observed significant increase of esculetin, luteolin and apigenin aglycones in the treated infusion suggest that the hesperidinase was able to remove glucose from esculin (esculetin 6-O-glucoside), luteoloside (luteolin 7-O-glucoside), and apigenin derivatives (apigenin-7-O-glucoside and apigenin-7-apioglucoside). Incubation with the enzyme combination enabled efficient hydrolysis of rutinosides and hesperidoside from diosmin, quercetin and kaempferol. The DPPH**•** scavenging activity of chamomile aqueous infusion can be attributed in part to flavonoids and possibly to some other polyphenols with low molecular weights.

Apigenin, the most abundant flavenoid, is recognised as one of the most bioactive phenolic compounds in chamomile. In comparison to its bound forms, which include mostly apigenin-7-O-β-glucoside and various acylated forms, the aglycone is endowed with much higher bioactivity. Li et al.^31^ showed that inhibitory abilities of individual flavonoids against amylase and glucosidase were in exactly the same sequence (apigenin > baicalein > scutellarin > chrysin > apigenin-7-O-glucuronide > baicalin > chrysin-7-O-glucuronide > isocarthamidin-7-O-glucuronide). The apigenin possessed the strongest enzyme inhibitory effect which was attributed to seven double bonds in the two aromatic rings and hydroxyls present on C-7 and C-4′ of ring B.

Isoquercetin showed α-glucosidase inhibition toward maltase and sucrase with IC_50_ values of 64.1 and 42.5 µM, respectively, which were 10 and 5 times, respectively, more potent than its corresponding diglucoside (quercetin-3-O-β-glucopyranosyl-glucoside). This observation suggested that the increase in hydrophilicity by the extra glucose moiety in glycosylated quercetin dramatically reduced the inhibitory effect^32^. Another report by Zhang et al.^28^ pointed to the flavonols (including kaempferol and quercetin glycoside), but not the flavanols (including catechin/epicatechin-glucoside and procyanidin dimer), as the most important contributors to the inhibitory activities against α-glucosidase and pancreatic lipase. Interestingly, and more against the latter, the Galloyl moiety of the flavanols has been considered as a prerequisite feature for the lipase inhibition^10^.

In a recent study, the ethanolic extract of *Chamomilla recutita* inhibited lipase activity (86.6 ± 0.3%) in addition to having high antioxidant and anti-glycation capacities^33^. While EC_50_ values have shown the potency of the natural compound, more valuable information can be obtained from the kinetics of inhibition by individual compounds or a mixture of compounds from the natural extract. The components of chamomile showed a non-competitive inhibition on glucosidase activity and a mixed inhibition on lipase activity (Figure 2). When an inhibitor binds to the enzyme and/or enzyme–substrate (ES) complex it is defined as non-competitive inhibition, in which the inhibitor affects only *V*_max_ of the reaction, but has no effect on ES complex formation. Mixed inhibition occurs when the inhibitor binds at a distinct site from the active site, but with simultaneous formation of an enzyme–inhibitor (EI) complex in a competitive manner and an enzyme–substrate–inhibitor (ESI) complex in a non-competitive way. Probably this result was due to the mixture of compounds found in the chamomile infusion containing compounds with both types of inhibition, as well as the high efficiency of the bioconversion reaction, which led to the conversion of active compounds to even more active metabolites that can act as enzyme inhibitors. Gholamhoseinian et al.^34^ found similar results showing that components of *Levisticum officinale* can bind to the enzyme or ES complex, blocking the pancreatic lipase activity.

Though a number of polyphenols have been reported as inhibitory to the action of digestive enzymes *in vitro*, only a limited number of reports have diagnosed their mode of inhibition, showing, however, contradictory results. For instance, phenolics from the methanolic extract of finger millet seed coat showed strong inhibition towards glucosidase and pancreatic amylase with non-competitive inhibition^35^, while phenolic acids from potato cultivars acted as effective mixed inhibitors for α-amylase and α-glucosidase and non-competitive inhibitors for aldose reductase^36^. Main polyphenols in yerba maté (chlorogenic acids, caffeoylquinic acids, dicaffeoylquinic acids) competitively inhibited pancreatic lipase activity in a concentration-dependent manner with an IC_50_ value of 1.5 mg MT/ml^8^. Such a variation in inhibitor potency, as well as the mode of enzyme inhibition, is not unusual since the inhibitor potency of polyphenolic compounds depends on several factors, such as the structure and stability of the inhibitors.

That finding is in agreement with an earlier study that showed the inhibitory effect of flavonoids on pancreatic lipase activity and reported that the presence of sugar units in flavonoid structures reduces their inhibitory power so that an aglycone form, such as quercetin, is a more efficient inhibitor than the glycosylated form, rutin^11^. Sergent et al.^37^ also reported that the phenolic compounds, including quercetin and kaempferol, exerted strong inhibitory activity on pancreatic lipase. *K*_m_ values for pancreatic lipase inhibition by quercetin were previously reported and varied from 2.15 ± 0.40 µM^11^ to 10 µM^38^. It is difficult to establish comparisons between the results reported by the above authors, since they were determined with different substrates/flavonoids as well as under different conditions of temperature and pH.

On the other hand, Zhang et al.^28^ analysed the difference between flavonol glycosides and their aglycones from lentil (Lens cultivars) cultivar extracts against pancreatic lipase activity, and they did not find statistically different values for each flavonoid pair. In a similar study, Li et al.^39^ evaluated the effect of a glucoside substituent in position 3 of the C ring of quercetin, analysing quercetin, isoquercetin and rutin. They observed that rutin, which possesses a disaccharide moiety, was the best pancreatic lipase inhibitor, followed by isoquercetin (monosaccharide moiety) and finally quercetin (aglycone). Those authors found that the placement of a double glycosylation of rutinoside for rutin, provided a higher possibility to interact with the enzyme by increasing the polarity of the pancreatic lipase–protein adduct, by hydrogen bonding formation, and by decreasing the hydrophobic environment near the catalytic site, necessary to hydrolyse the triacylglycerols.

## Conclusion

*Matricaria chamomilla* L. is considered a good source of functional polyphenolic compounds and the enzymatic treatment of flavonoids is known to improve bioactivity of such compounds due to removal of sugar molecules. In this study, we observed that around 30 putatively identified compounds were modified after treatment of chamomile infusion with hesperidinase and β-galactosidase. The infusion showed interesting results for the inhibition of digestive enzymes, mainly on pancreatic lipase. Thus, the enzymatic treatment of polyphenols can lead to bioactivity enhancement, showing the good potential of the enzymatically modified chamomile infusion as a functional food for human health benefits.
